# Supplementary figures and images for: Immunization with tegument nucleotidases associated with a subcurative praziquantel treatment reduces worm burden following Schistosoma mansoni challenge
Source: PeerJ. 2013 Apr 2;1:e58. doi: 10.7717/peerj.58 (PMC3628383; doi:10.7717/peerj.58)

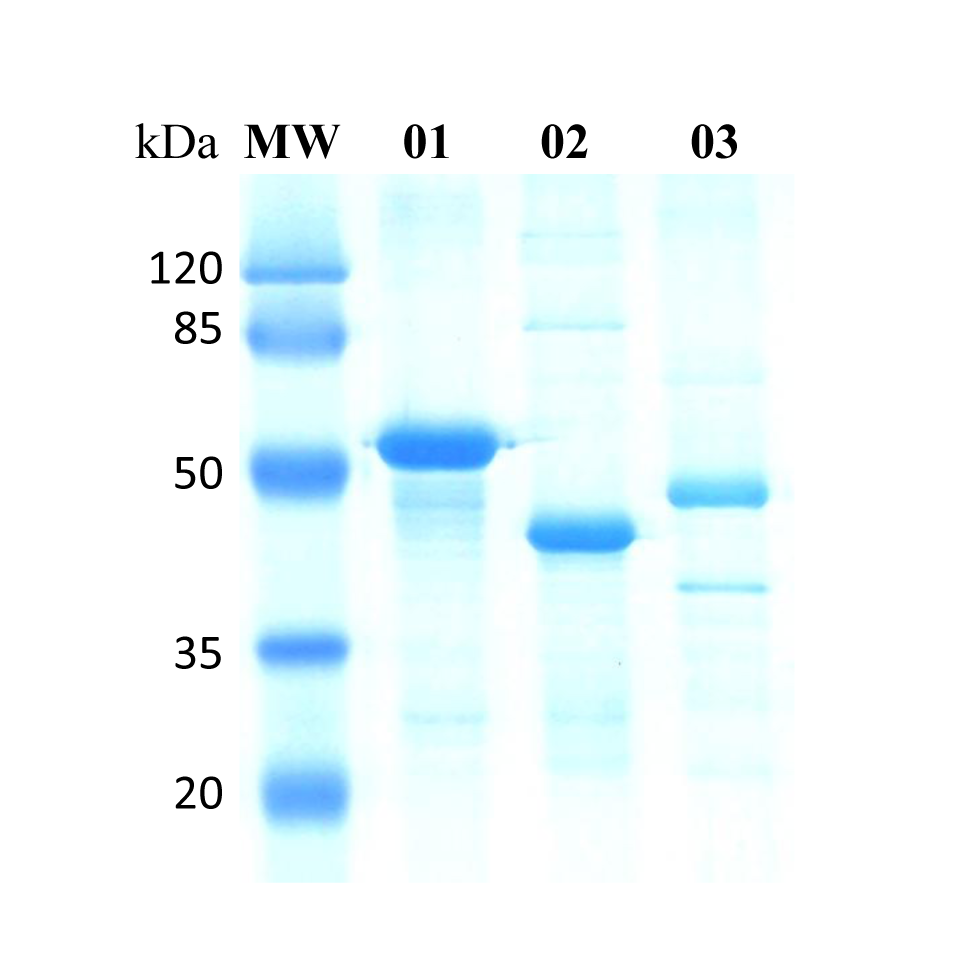

Supplement: Fig. S1 — MW – Protein molecular weight marker: 120, 85, 50, 35 and 20 kDa. 01 – SmAP; 02 – SmNPP-5; 03 – SmNTPDase. [file peerj-01-58-s001.png]

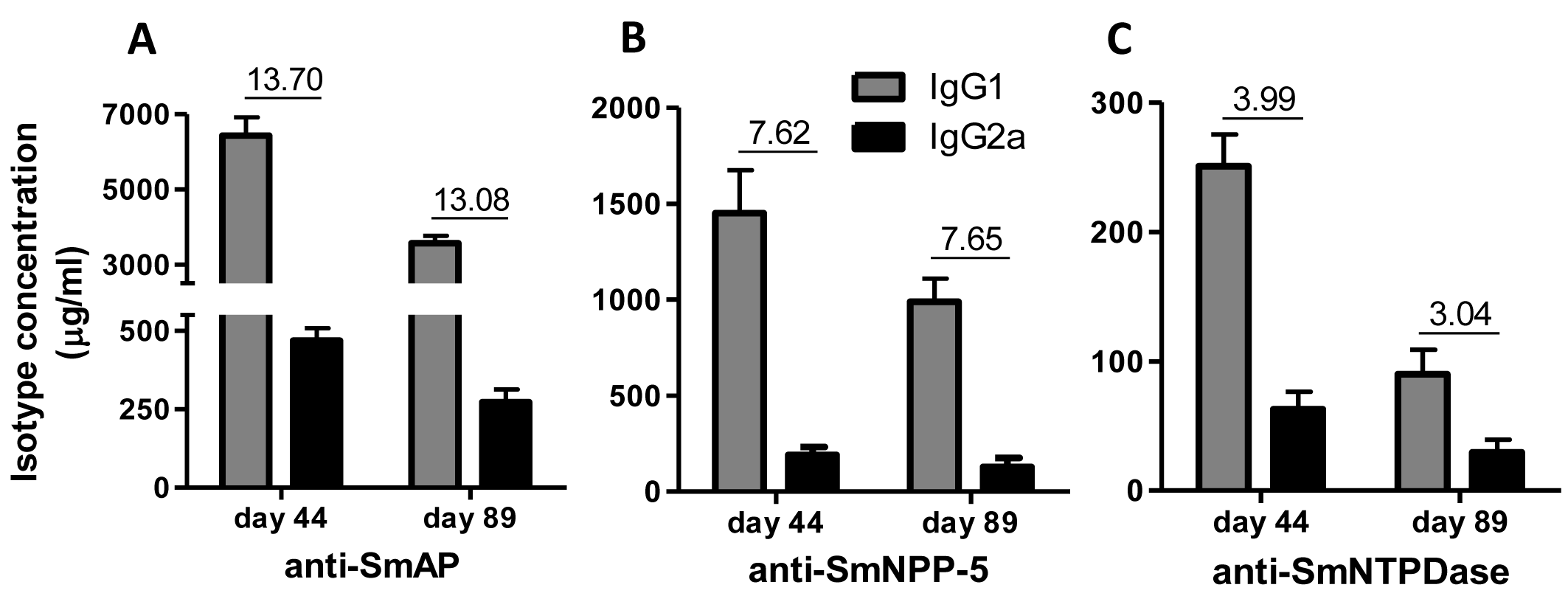

Supplement: Fig. S2 — (A) Specific IgG1 and IgG2a levels against SmAP before and after challenge. (B) Specific IgG1 and IgG2a levels against SmNPP-5 before and after challenge. (C) Specific IgG1 and IgG2a levels against SmNTPDse before and after challenge. The bars are Mean ± SEM. The numbers over the bars are the IgG1/IgG2a ratios. [file peerj-01-58-s002.png]

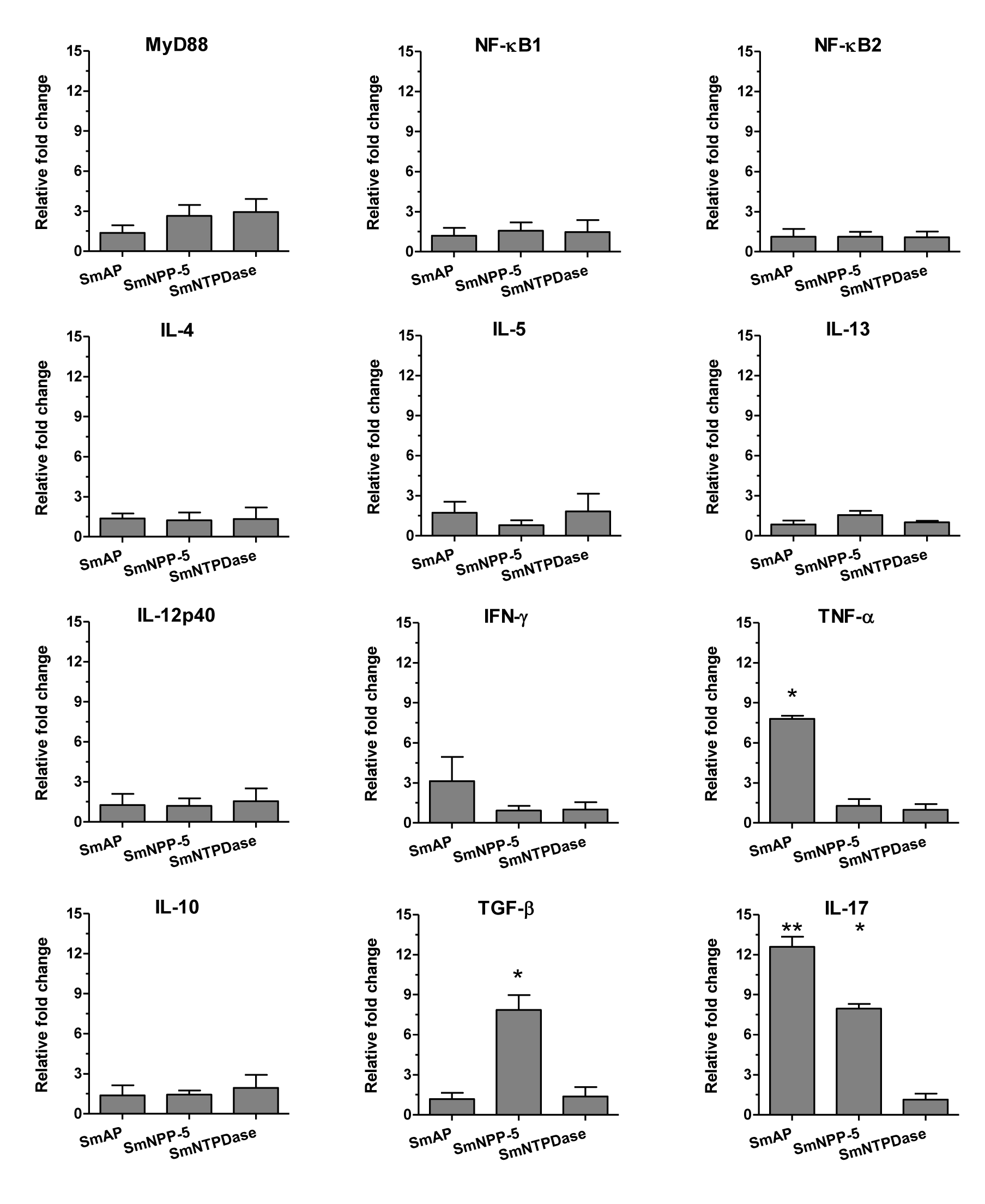

Supplement: Fig. S3 — The bars are Mean ± SEM; ∗ = ρ ≤ 0.05. [file peerj-01-58-s003.png]

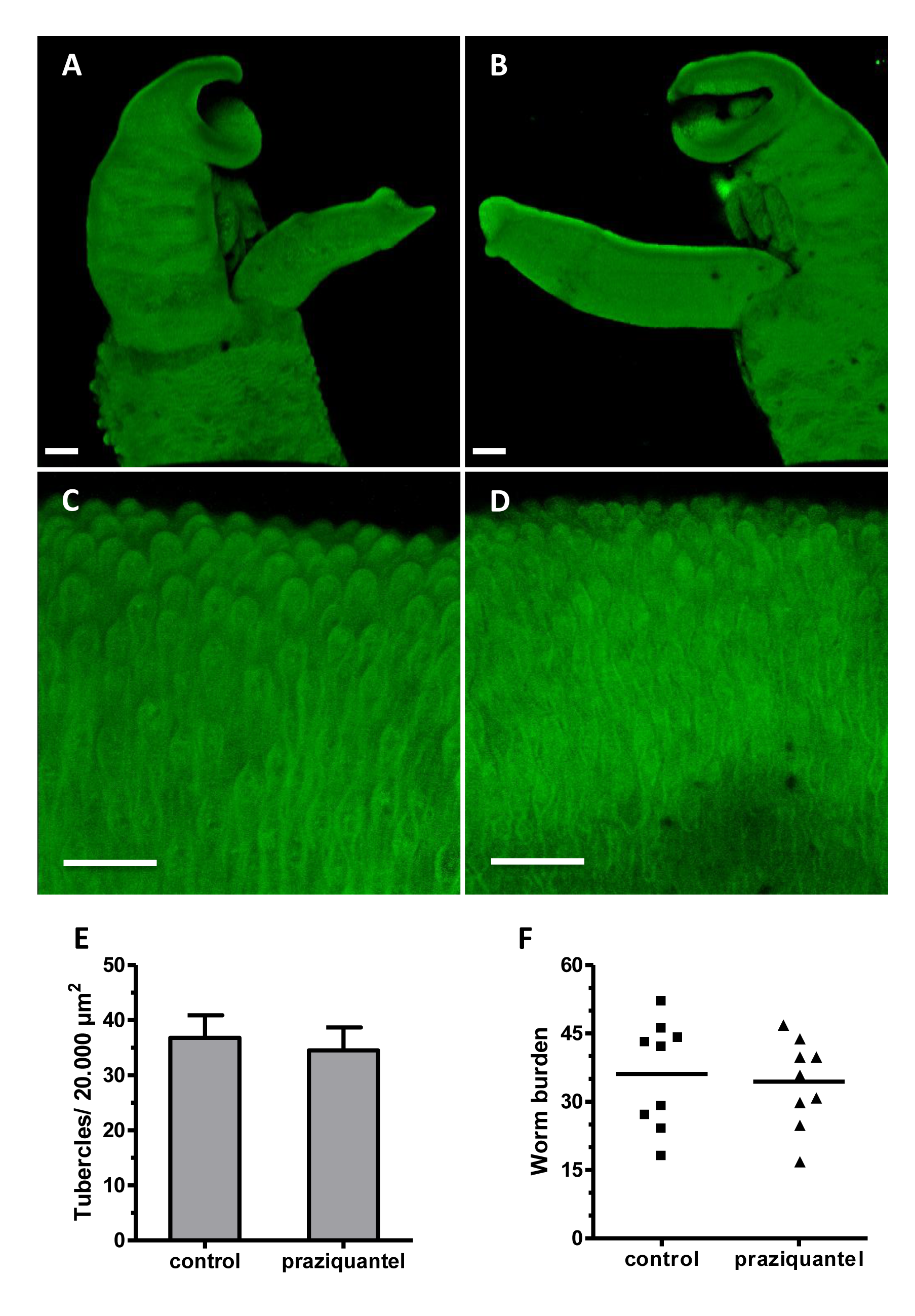

Supplement: Fig. S4 — (A) and (B) Worms morphology analysis by confocal microscopy of worms perfused 2 h after second dose of Praziquantel from control and treated mice, respectively. (C) and (D) Tegumental tubercles analysis by confocal microscopy of worms perfused 2 h after second dose of Praziquantel from control and treated mice, respectively. (E) Tegumental tubercles number from worms perfused 2 h after second dose of Praziquantel treatment; the bars are the Mean ± SD. (E) Worm burden dispersion of mice perfused 45 days after infection treated or not with subcurative doses of Praziquantel; the lines represent the Means. [file peerj-01-58-s004.png]

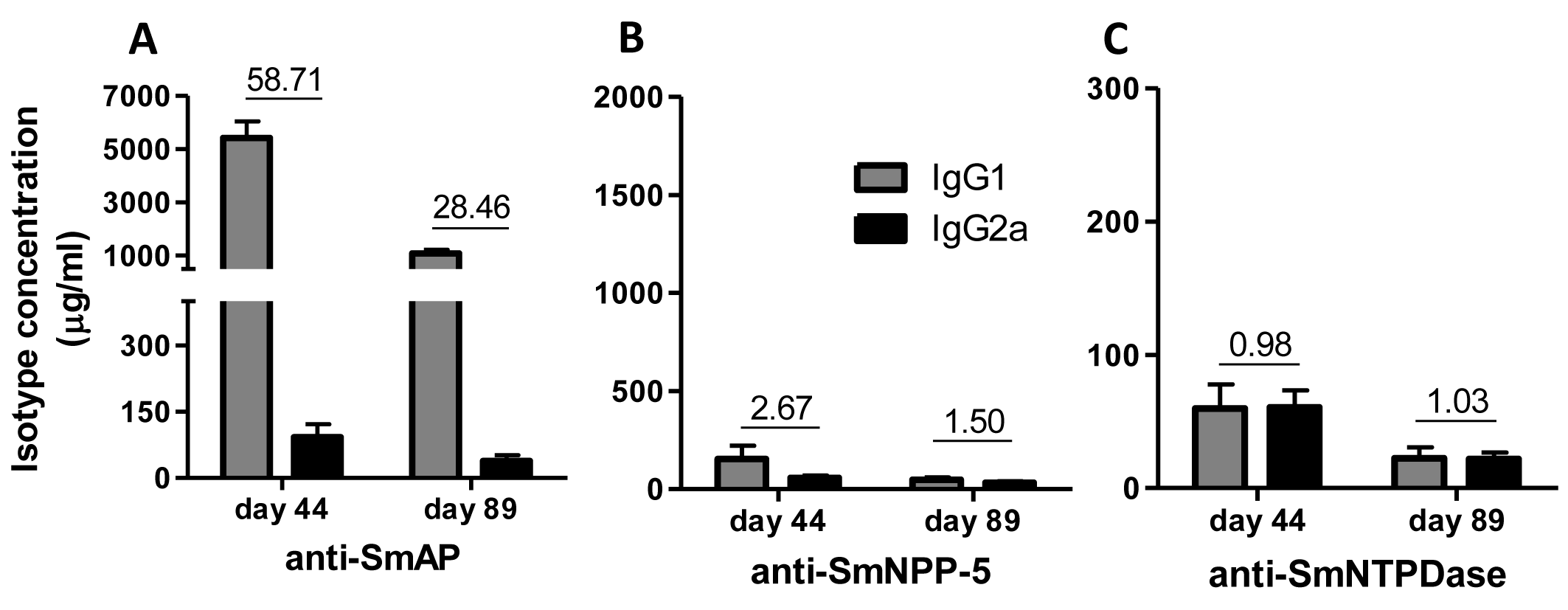

Supplement: Fig. S5 — (A) Specific IgG1 and IgG2a levels against SmAP before challenge and after Praziquantel treatment. (B) Specific IgG1 and IgG2a levels against SmNPP-5 before challenge and after Praziquantel treatment. (C) IgG1 and IgG2a levels against SmNTPDse before challenge and after Praziquantel treatment. The bars are Mean ± SEM. The numbers over the bars are the IgG1/IgG2a ratios. [file peerj-01-58-s005.png]
